# Influence of gold, silver and gold–silver alloy nanoparticles on germ cell function and embryo development

**DOI:** 10.3762/bjnano.6.66

**Published:** 2015-03-05

**Authors:** Ulrike Taylor, Daniela Tiedemann, Christoph Rehbock, Wilfried A Kues, Stephan Barcikowski, Detlef Rath

**Affiliations:** 1 Institute of Farm Animal Genetics, Friedrich-Loeffler-Institut, Federal Research Institute of Animal Health, Hoeltystrasse 10, 31535 Mariensee, Germany; 2 Technical Chemistry I and Center for Nanointegration Duisburg-Essen (CENIDE), University of Duisburg-Essen, Universitätsstrasse 7, 45141 Essen, Germany

**Keywords:** bimetallic nanoparticles, nano gold, nano silver, ontogenesis, oocyte, reprotoxicity, spermatozoa

## Abstract

The use of engineered nanoparticles has risen exponentially over the last decade. Applications are manifold and include utilisation in industrial goods as well as medical and consumer products. Gold and silver nanoparticles play an important role in the current increase of nanoparticle usage. However, our understanding concerning possible side effects of this increased exposure to particles, which are frequently in the same size regime as medium sized biomolecules and accessorily possess highly active surfaces, is still incomplete. That particularly applies to reproductive aspects, were defects can be passed onto following generations. This review gives a brief overview of the most recent findings concerning reprotoxicological effects. The here presented data elucidate how composition, size and surface modification of nanoparticles influence viablility and functionality of reproduction relevant cells derived from various animal models. While in vitro cultured embryos displayed no toxic effects after the microinjection of gold and silver nanoparticles, sperm fertility parameters deteriorated after co-incubation with ligand free gold nanoparticles. However, the effect could be alleviated by bio-coating the nanoparticles, which even applies to silver and silver-rich alloy nanoparticles. The most sensitive test system appeared to be in vitro oocyte maturation showing a dose-dependent response towards protein (BSA) coated gold–silver alloy and silver nanoparticles leading up to complete arrest of maturation. Recent biodistribution studies confirmed that nanoparticles gain access to the ovaries and also penetrate the blood–testis and placental barrier. Thus, the design of nanoparticles with increased biosafety is highly relevant for biomedical applications.

## Review

### Reprotoxicology

Repotoxicological studies are a mandatory part during every stage of drug approval processes. They are of paramount importance as possible defects may not only affect the person or animal directly treated with the drug but possible adverse effects may also be predominantly relevant for following generations. This does not only apply to conventional drugs, but also to nanoparticles. In a multigenerational study using gold nanoparticles in an *caenorhabditis elegans* model it was recently shown that after oral nanoparticle exposure reproduction rate was clearly affected in the F2 generation [1]. While many reprotoxicological examinations are performed as clinical or animal trials, there is also a wide field of in vitro studies. From the final stages of gamete maturation up to the blastocyst stage, when implantation becomes imminent, the reproductive process can be monitored employing easily obtainable, well-defined primary cells with clearly defined functions by using internationally standardized protocols. The period around conception is characterized by considerable cytological and molecular restructuring and is therefore particularly sensitive to disturbances. Hence, otherwise subtle effects can be detected more easily. Furthermore, oocytes and spermatozoa possess very different features regarding metabolic activity [2], membrane composition [3–4], and compartmentalization [5], making it possible to observe in how far such properties influence sensitivity towards potentially toxic substances. This facilitates in vitro tests which significantly surpass standard assays traditionally used in somatic cell culture. Table 1 gives examples of common reprotoxicological in vitro tests and their predictive values. However, despite its obvious importance, reprotoxicological testing of nanoparticles has so far been frequently neglected. While in the last two years approximately 1000 articles per year were published concerning nanotoxicology in general, only 3 of them concerned spermatozoa and 2 oocytes (source: web of knowledge). We are therefore unable to draw a comprehensive picture of nanoparticle reprotoxicology. However, studies concerning gold (AuNP) and silver nanoparticles (AgNP) are relatively well represented in current literature and shall be reviewed in the following chapters. Furthermore, the actual relevance of gold and silver nanoparticles in terms of exposure and distribution to reproductive relevant sites will be highlighted.

**Table 1:** Common reprotoxicological in vitro tests and their predictive value.^a^

cell type	test	tools	parameter description	biological importance

Spermatozoa [6]	Membrane integrity [7]	Live/Dead stain, Flowcytometer	Percentage of membrane impaired, i.e., dead spermatozoa	An intact membrane ensures the existence of receptors necessary for binding to the oocyte
	Motility [8]	Computer assisted sperm analysis	Percentage of total motile spermatozoa and progressive motile spermatozoa	Sperm need to actively swim to the oocyte
	Morphology [9]	Phase contrast microscope	Percentage of spermatozoa with normal morphology	Abnormal morphology often leads to inability to move, bind to oocyte, or fertilize
	IVF: oocyte penetration	Phase contrast microscope	Ability of sperm to penetrate the oocyte	Spermatozoa need to bind to the *Zona pellucida* and enter the oocyte for fertilization
	IVF: Pronucleus formation	Nuclear stain, phase contrast microscope	Ability within the for IVF deployed sperm population to decondens the sperm nuclear DNA	Sperm tail is released and nucleus decondenses in order to fuse with female pronucleus
Oocyte [10]	IVM: Metaphase plate development	Nuclear stain, phase contrast microscope	Percentage of oocytes having reached the metaphase of the second meiosis and excluded a polar body	Nuclear maturation is vital for fertilization and further development
	IVM: cumulus cell expansion	Stereo microscope	Rating how far the cumulus cells moved apart and away from the oocyte during maturation	Cumulus cell layer needs to expand in order to stop the meiotic arrest of the oocyte
	IVF: sperm/oocyte	Phase contrast microscope	Number of sperm which have penetrated the oocyte	Oocyte is responsible for blocking all other sperm after the first one entered
	IVF: Pronucleus formation	Nuclear stain, phase contrast microscope	Decondensation of the oocytes’ nucleus into the female pronucleus induced by fertilization	After fertilization the oocytes´ nucleus has to exclude the second polar body and form the female pronucleus to fuse with the males´
Embryo [11]	IVC: Cleavage rate	Stereo microscope	Number of embryos that show at least two blastomeres and time required for cell division	Cell division in early embryo to first form the morula and later the blastocyst
	IVC: Blastocyst rate	Stereo microscope	Number of embryos having reached blastocyst stage	Blastocyst rate indicates cell quality and quality of culture conditions
	IVC: Blastocyst cell number	Nuclear stain, fluorescence microscope	Number of blastomeres per blastocyst	Blastocysts need to have sufficient cell number in order to develop further

^a^IVF: in vitro fertilisation; IVM: in vitro maturation; IVC: in vitro culture.

### Gold and silver nanoparticles – exposure and biodistribution

Exposure to man-made nanosized particles is not a recent event. As diesel fumes or in the form of air-borne particles released during welding, humans have been unintentially confronted with such materials for several decades. However, in the past twenty years technology has evolved sufficiently to allow for the mass production of engineered nanoparticles. Due to their fascinating optical, chemical and physical properties they quickly found their way into many products. With regard to gold nanoparticles this applies particularly to biomedical purposes such as cancer imaging and therapy as well as drug delivery [12–14], but also for analytical applications [15] and nanoelectronics [16]. Silver nanoparticles have been developed for catalysis [17], optics [18] and electronics [19], but they are mainly employed in the medical sector and in consumer products for their antimicrobial properties [20–21]. This variety in applications generates several potential exposure routes for gold and silver nanoparticles, including injection and inhalation particularly for biomedical applications, but also ingestion and skin contact for medical and consumer products. The uptake behaviour of nanoparticles differs depending on the mode of exposure. If exposure occurs by inhalation the majority of particles is cleared from the lungs by macrophage-mediated transport of particles to the airways and subsequent mucociliary escalation to larynx and pharynx. But there is still a substantial amount of particles translocated across the air–blood barrier [22]. The magnitude of particle transfere is inversely correlated to particle size [23]. In contrast, particle uptake following dermal exposure has so far not been observed as nanoparticles do not penetrate beyond the most superficial skin layers [24–25]. On the other hand, uptake via ingestion has been proven for silver [26–28] as well as gold nanoparticles [29–30]. Interestingly, for AgNP, it has been suggested that mainly ionic silver, released from the actual particles due to dissolution is absorbed via the intestinal tract, followed by an in vivo formation of silver salts like AgS, AgSe and AgCl [28]. Gold nanoparticles showed a size-dependent intestinal absorption while small (<5 nm) particles are preferably taken up [29]. However, in either case uptake via ingestion remained at a very low level with <1% for AuNP [29] and <0.1% for AgNP [28] of the given dose. Once nanoparticles entered the body, the biodistribution depends on factors like particle size [31–32] and surface functionalization [33]. No clear trends have been established yet as to how those factors determine the biodistribution of the particles and further aspects like nanoparticle concentration, animal species, strain, age, breeding, housing facilities or inter-animal interaction have been suggested to have an impact on the final outcome [28]. However, regardless of the various nanoparticle properties or other circumstances the foremost target organ of gold and silver nanoparticles seems to be the liver followed by spleen and kidney [34–35]. But particles have also been localized in other organs including brain and testis, which represent sites particularly protected by the blood–brain and the blood–testis barrier [22,28,31–32,36–37]. An interesting finding, especially under reprotoxicological aspects, is that several studies consistently noted an accumulation of AuNP as well as AgNP over time in the testis [27–28,37]. At least for AuNP, actual crossing of the blood–testis barrier has also been shown, though no detrimental effects on fertility were found [38]. A study examining the influence of AgNP on spermatological parameters following intraveneous injection revealed a reduced sperm count and an increase in sperm DNA damage [39]. It remained unclear though whether AgNP had actually reached the germinative tissue, or whether the effect was caused by silver ions. However, it supports findings made on spermatogonial stem cells in vitro, which claimed a decrease in cell proliferation after AgNP exposure [40–41]. Observations concerning female reproductive organs are rather rare as most nanoparticle biodistribution studies have been performed solely on male animals or in case females were used ovaries or uteri were not examined. However, while in one study no nanoparticles where found in either ovary or placenta after intraveneous injection of AuNP [42], further studies reported the detection of AuNP after intertracheal or intravenous application in placenta and fetus [43–44] as well as uterus [45]. In the first study electron microscopy was chosen for the examinations, which is perhaps not the most effective screening method. In the latter cases the AuNP were either radio-labelled and detected by gamma-spectroscopy or analyzed through hard X-ray microfocus beamline synchrotron imaging which both represent very sensitive detection methods. Silver nanoparticles have been observed to enter the ovaries [27] as well as passing through placenta and breast milk [46] in quantities comparable to the loads found in liver and blood after oral administration. Transplacental transfer to mouse embryos after intraveneous application of nanoparticles to the dam was also confirmed for AgNP [47]. Therefore, it seems reasonable to assume that they are as much if not more targeted by nanoparticles than their male equivalents as ovaries for instance are not protected by a biological barrier. Interestingly, a recent study revealed no adverse effects to the reproductive process after oral administration to rats [48].

In summary, the listed results emphasize the importance of reprotoxicological testing of nanoparticles, as close and potentially detrimental contact to developing germ cells and embryos must be presumed a realistic scenario.

**Figure 1:**
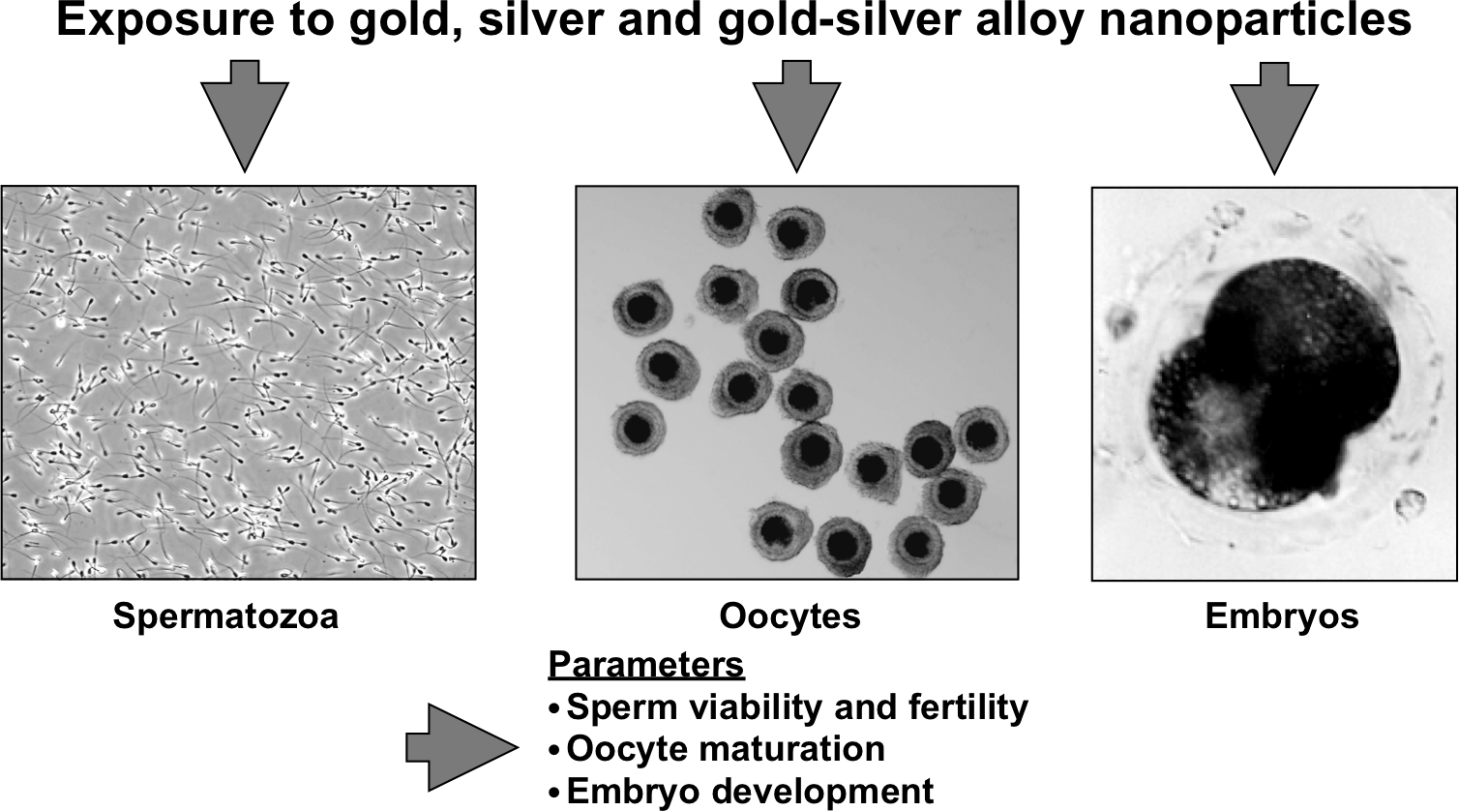
Schematic representation of experiments conducted within the collaboration project REPROTOX.

**Figure 2:**
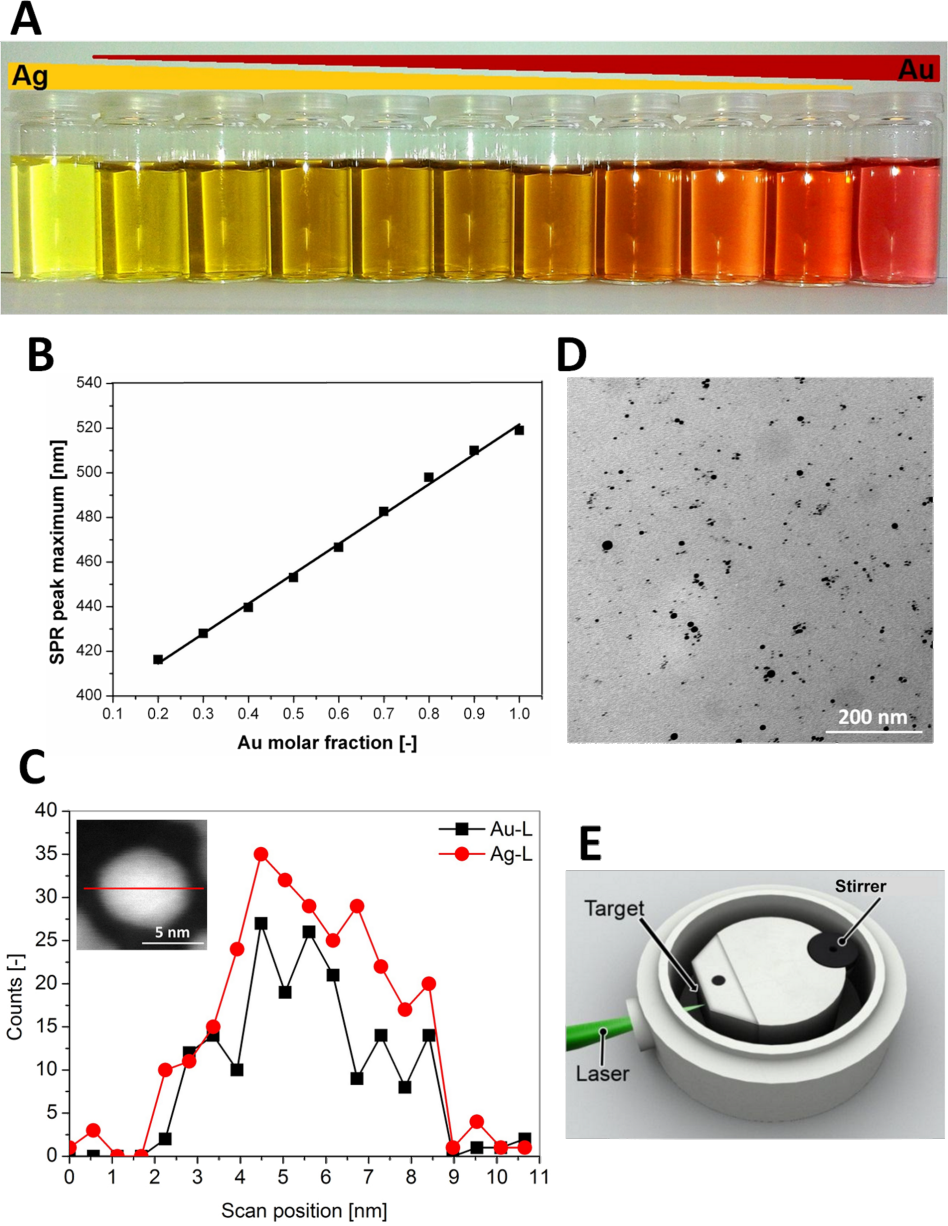
(A) Exemplary AuAg colloids with different molar fractions. (B) Correlation of gold molar fraction with the maximum surface plasmon resonance extinction peak. (C) TEM-EDX line scan with inset showing high-angular annular dark field micrograph. (D) TEM micrograph of a Ag50Au50 nanoparticle dispersion after stabilisation with BSA. (E) Aluminium batch chamber for the synthesis of silver and gold–silver alloy nanoparticles. Reproduced with permission from [50]. Copyright 2014 Royal Society of Chemistry.

The following paragraphs focus on the discussion of data obtained by the collaboration project REPROTOX, which formed part of the German Research Foundations priority program 1313 “Biological responses to nanoscale particles”. The aim was to study the impact of gold and silver nanoparticles, and to a degree gold–silver alloy nanoparticles, on spermatozoa, oocytes and embryos in vitro using murine as well as bovine and porcine models (Figure 1) [49–51]. The nanoparticles used in our studies where produced by laser ablation in liquids [52–53], which allows for the synthesis of highly pure particles free of any stabilisers or reducing agents which might exert a toxicological impact of their own. In addition, laser ablation of solid noble metal alloy targets in water results in homogenous alloy nanoparticle colloids (Figure 2) [50,54]. Besides representing the collected data, it will be put into context with other recent studies on the same subject.

### Effect of gold and silver nanoparticles on spermatozoa

The main purpose of spermatozoa is the safe delivery of the male genome to the oocyte passing through a potentially harsh environment. Therefore male germ cells possess special attributes which accommodate for these conditions. The sperm nucleus, for instance, is surrounded by a tightly fitted double layered nuclear membrane, which is condensed to a degree that the DNA within almost reaches crystalline properties [55–56]. The mitochondria, producers of reactive oxygen species, are placed at the midpiece of the spermatozoon and thus spatially removed from the nucleus, which limits the affliction of oxidative damage to the precious cargo [57]. During the course of our studies another safety feature was noted: Compared to somatic cells which readily incorporate nanoparticles [58–60], the sperm plasma membrane, prior to acrosome reaction, seems literally impenetrable for any nanoparticles we tested which encompassed ligand-free AuNP (diameter 10.8 nm, Zeta potential −25 mV) and oligonucleotide conjugated AuNP (diameter 7.3 nm, 94 biomolecules per particle, Zeta potential −32 mV) which were tested by using bovine sperm, as well as bovine serum albumin (BSA) coated gold (diameter 6–20 nm), silver (diameter 11 nm; AgNP) and various gold silver alloy nanoparticles (silver molar fraction 20, 50 and 80%; diameter 6–7 nm; AuAgNP) where trials were conducted with porcine spermatozoa [49–50]. This observation does not entirely agree with what can be found in literature. Internalisation of nanoparticles made from gold [61–62] or other materials like Fe_3_O_4_–PVA [63–64], Eu_2_O_3_, PVP–Eu(OH)_3_ NP, PVA–Eu(OH)_3_ [65] and bio-conjugated CdSe/ZnS quantum dots [66] into spermatozoa has been reported. However, the picture evidence provided to support these claims does not withstand critical examination at least with regard to intact, non-acrosome reacted spermatozoa, which are the ones mattering for fertilisation. The fact that it may be difficult for nanoparticles to enter into these highly specialised cells does not seem unreasonable. Nanoparticles have been observed to enter into somatic cells mostly via endocytosis [59–60], a cellular mechanism which sperm do not possess. Additionally, membrane wrapping, which has been described as an important step in nanoparticle uptake [67] cannot be performed by spermatozoa due to their rather rigid and tensely streched plasma membrane.

**Figure 3:**
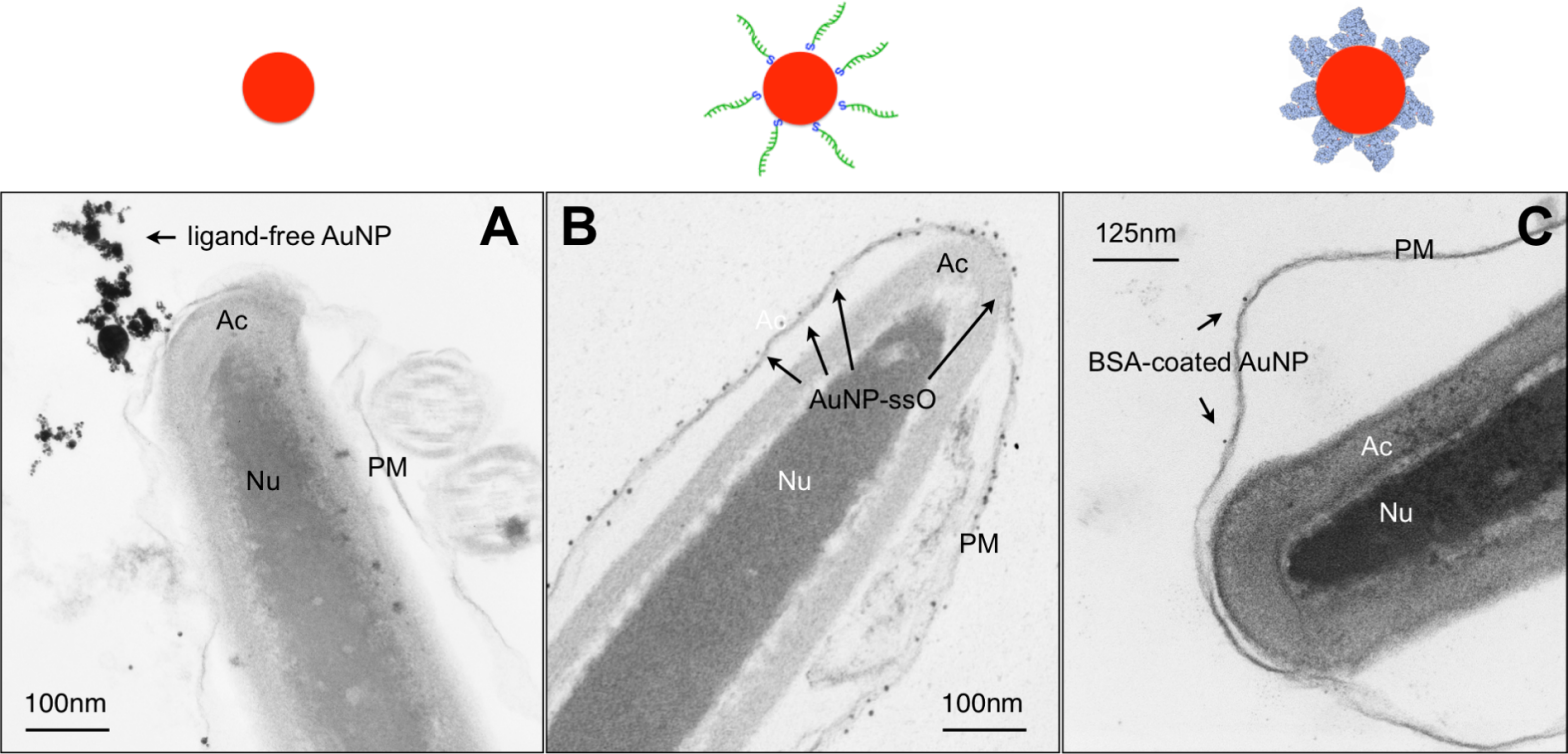
Representative TEM-micrographs of bovine spermatozoa after co-incubation with gold nanoparticles (AuNP) (10 µg/mL Au) for 2 h at 37 °C. (A) Ligand-free AuNP, (B) oligonucleotide-conjugated AuNP, (C) BSA-coated AuNP. Arrows point out AuNP. Inserts depict the displayed sperm section in total. Above each section the relevant nanoparticle type is displayed schematically. PM = plasma membrane; Ac = acrosome; Nu = nucleus (adapted from [49–50]).

Another noteworthy point is the variation observed in our experiments concerning nanoparticle attachement to spermatozoa, which seemed to be driven by the surface modifications. While ligand-free AuNP and gold nanoparticles conjugated to single stranded oligonucleotides (AuNP–ssO) at least attached to the sperm membrane, BSA-coating of nanoparticles prevented even such an attachment (Figure 3). The ligand-free AuNP and the AuNP–ssO were co-incubated with spermatozoa in a medium consisting only of inorganic salts and fructose without any protein source. Even though ligand-free gold nanoparticles possess a negative net-charge they predominantly have an uncharged metal surface prone to interact with electron donors such as thiol groups found on the negatively-charged sperm membrane [57,68]. Oligonucleotide-conjugated nanoparticles might bind directly to nucleic acid specific binding sites which have been shown to exist on the sperm surface [69]. The fact that BSA coating inhibits nanoparticle adsorption to the sperm membrane seems surprising though, since albumins have been described to adsorb to the sperm membrane [70]. As the adsorption of albumins to the surface of gold nanoparticles can induce conformational changes though [71], the surface moieties diplayed by the BSA–gold nanoparticle complexes might be different to native BSA and thus leading to unexpected interaction patterns.

The difference in nanoparticle attachment to spermatozoa observed in our studies also had an impact on the toxic effect the particles exerted. Co-incubation with ligand-free AuNP and AuNP–ssO at a concentration of 10 µg/mL lead to a decrease in sperm motility (Table 2) [49], which is an effect documented also by other authors for gold nanoparticles [61–62].

**Table 2:** Sperm viability parameters after co-incubation of bovine sperm with ligand-free or oligonucleotide-conjugated AuNP for 2 h at 37 °C [49].

AuNP Concentration µg/mL	AuNP type	% motile sperm	% membrane intact sperm	% normal morphology

0		54.3	79.5	83.8
0.1	ssO conjugated^a^	52.6	79.4	86.8
0.1	ligand-free	54.9	77.9	86.6
1	ssO-conjugated^a^	51.7	78.9	83.3
1	ligand-free	47.5	79.5	85.8
10	ssO-conjugated^a^	43.1^b^	80.7	84.7
10	ligand-free	32.2^b^	80.8	85.4

^a^ssO = single stranded oligonucleotide; ^b^*p* < 0.05.

**Figure 4:**
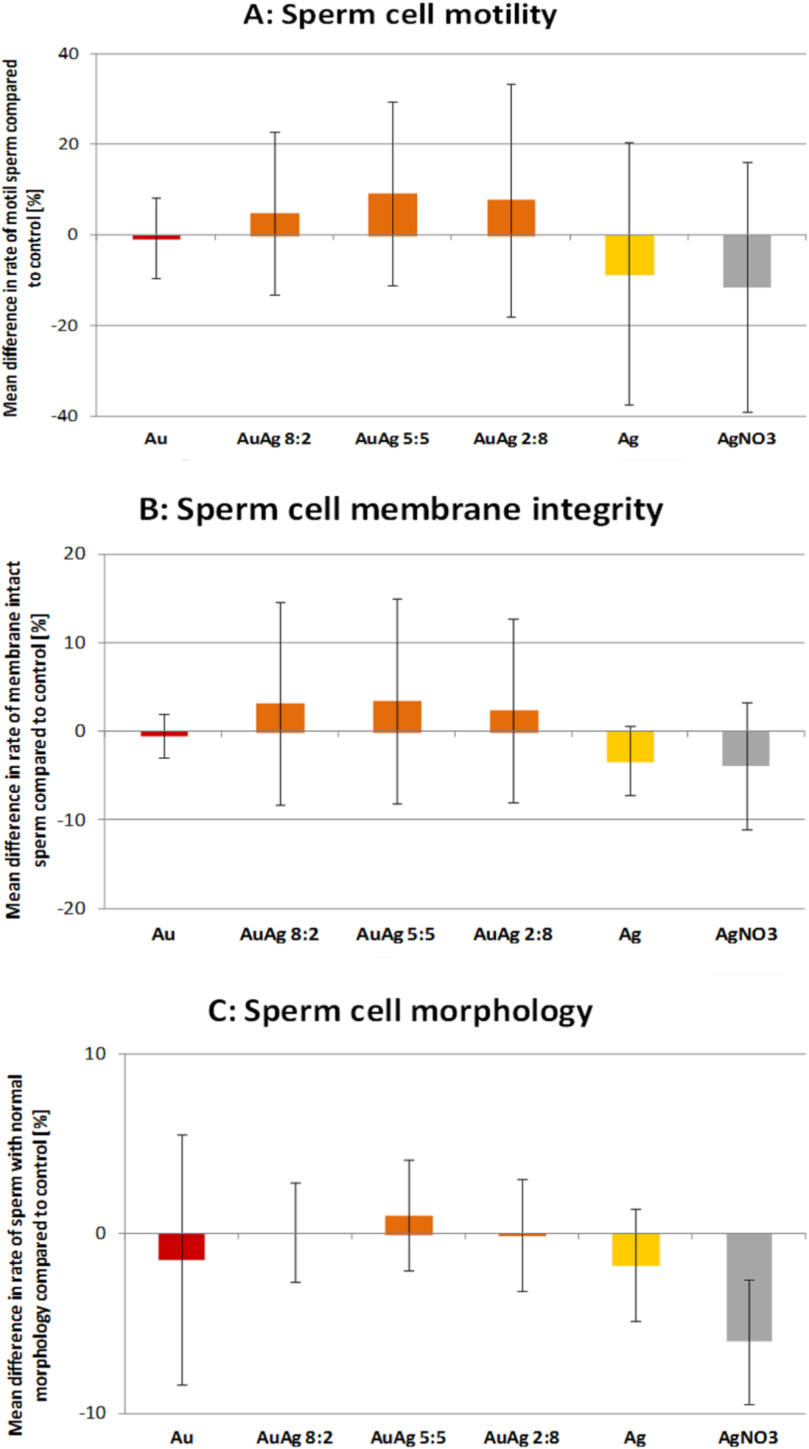
Sperm viability parameters after co-incubation of sperm for 2 h at 37 °C with various nanoparticle types and a silver nitrate control. Nanoparticle concentration was 10 µg/mL. (A) Motility assessed with Computer Assissted Sperm Analysis, (B) Membrane integrity assessed with propidium iodide stain and flow cytometer, (C) morphology assessed with phase contrast microscope and evaluation of 200 sperm cells per group per day. Shown are percentage of spermatozoa, which differ compared to the control [values are mean ± SD]. Reproduced with permission from [50]. Copyright 2014 Royal Society of Chemistry.

Interestingly, neither BSA-coated AuNP, AgNP or AuAgNP affected sperm motility (Figure 4) [50]. We hypothesized that membrane-attached gold nanoparticles may lead to a decrease in motility (i) either by production of reactive oxygen species (ROS) or (ii) by binding to free thiols present on the sperm surface as gold nanoparticles posses a high affinity to thiol groups. The thiols on the sperm surface are part of membrane bound Na^+^/K^+^-ATPases and Ca^2+^-ATPases, whose inhibition has been reported to induce a rapid loss in motility [57]. While increased ROS production in sperm due to nanoparticle exposure could not be confirmed, a decrease of free thiols on the sperm surface was noted, which indicates the validity of the second hypothesis [49]. It thus seems plausible that coating of gold nanoparticles with BSA alleviates the detrimental effect on motility as it prevents nanoparticle attachment to sperm. However, it does so even in case of nanoparticles containg silver, which are generally viewed as considerably more toxic than AuNP [35] and have also been noted to cause a drop in sperm motility [62]. Therefore, BSA-coating might be a suitable way to increase nanoparticle biocompatibility. Sperm membrane integrity and morphology, two further important sperm viability parameters, remained unaffected by any of the nanoparticle variations tested in our trials (Table 2, Figure 3). This does not confirm previously published findings, which reported grave morphological defects [61] as well as increased membrane damage [62] after exposure to AuNP. A direct comparison is difficult though as in case of the former study no concentration were given. In the latter the concentration indicating the onset of adverse effects was higher than in the experiments we conducted. Furthermore, both experiments were run using nanoparticles produced by chemical means, which could lead to residual stabilizing ligands remaining from synthesis, still present in the final solution, where they might cause cross contaminating effects.

In case of ligand-free as well as oligonucleotide conjugated AuNP we also conducted experiments testing not only sperm viability parameters after nanoparticle exposure but also fertilising potential including preimplantation development of the emerging embryos using in vitro fertilisation [49]. Interestingly, co-incubation of sperm with 10 µg/mL ligand-free AuNP led to a 50% decrease in the pronucleus development rate, a parameter indicating successful fertilisation of the oocyte. Oligonucleotide conjugated AuNP had no such effect. However, the development of the fertilised egg into the preimplantation embryo was not affected by exposure of spermatozoa to nanoparticles prior to fertilisation. The reason why contact to ligand-free AuNP affected the sperm fertilising potential in such a way has not been clarified yet and can only be speculated about. In contrast to AuNP–ssO which attach to sperm as primary particles due to their good electrosteric stability in salt containing media, ligand-free AuNP attach as agglomerates because their electrostatical stabilisation is shielded once exposed to salt ions (Figure 3). A possible explanation might be that those agglomerates geometrically interfere with oocyte–sperm interactions. Interestingly, a study investigating the effect of silver, titatium dioxide and cobalt nanoparticles on sea urchin sperm observed no impact on sperm fertilising capability, but developmental anomalies of the emerging embryos [72]. Future research needs to clarify the effect of nanoparticle exposure on the fertilising capability of spermatozoa, since it has proven to be a sensitive indicator of nanoparticle reprotoxicity. A valuable tool for such examinations could be recently developed transgenic pigs [73–74]. Their spermatozoa display a distinct green flourescence, which could be utilised in competitive fertilisations assays.

### Effect of gold and silver nanoparticles on oocytes

**Figure 5:**
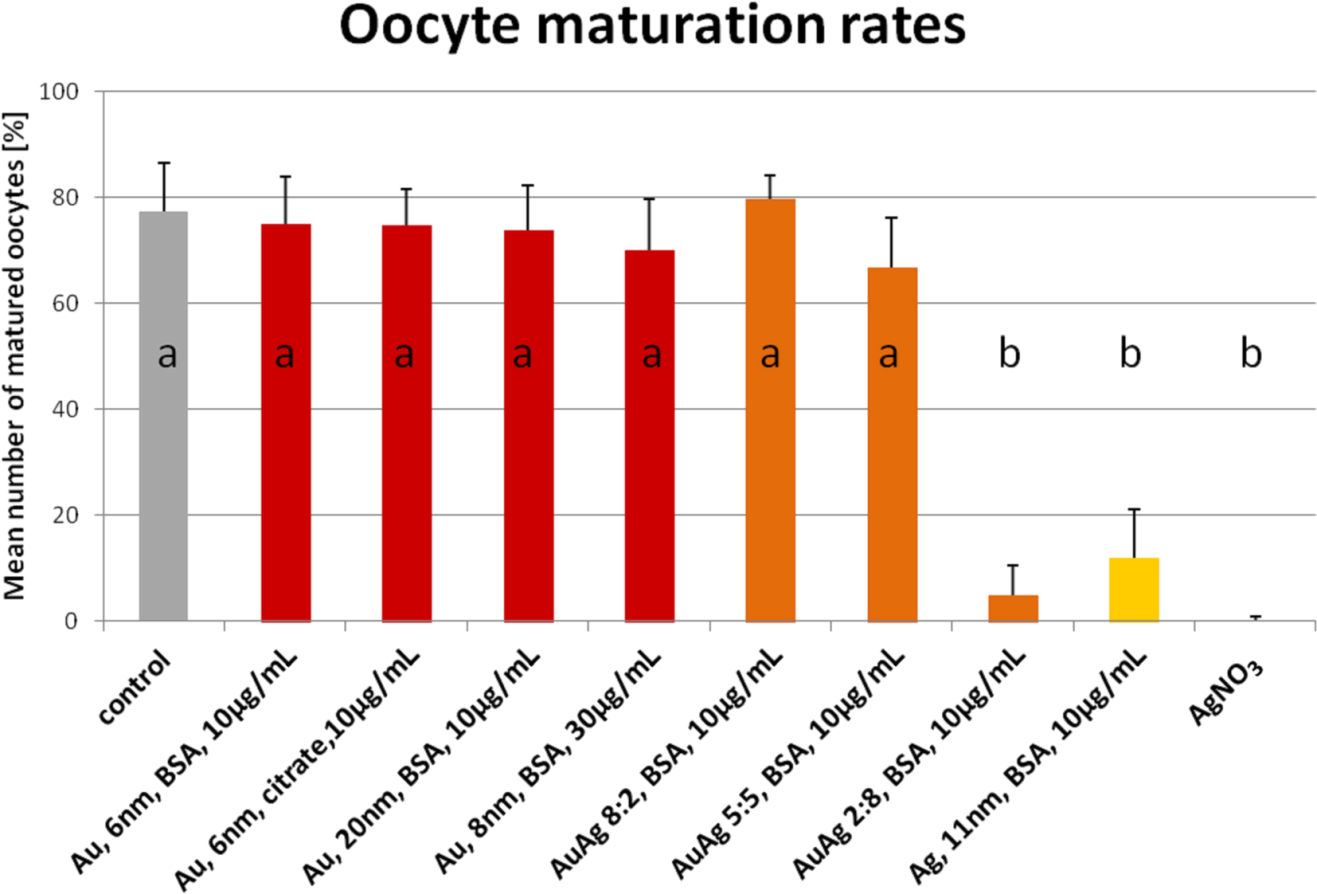
Oocyte maturation rates after 46 h of in vitro maturation in the presence of various nanoparticle types or silver nitrate in the maturation medium during the complete in vitro maturation time. Maturation rate defined in this case as percentage of oocytes displaying a metaphase plate and extruded polar body (second meiotic division) [values are mean ± SD; a,b *p* < 0.05]. 350 oocytes were assessed per group. Nanoparticle concentration was 10 µg/mL and all particles were conjugated with bovine serum albumin. Reproduced with permission from [50]. Copyright 2014 Royal Society of Chemistry.

**Figure 6:**
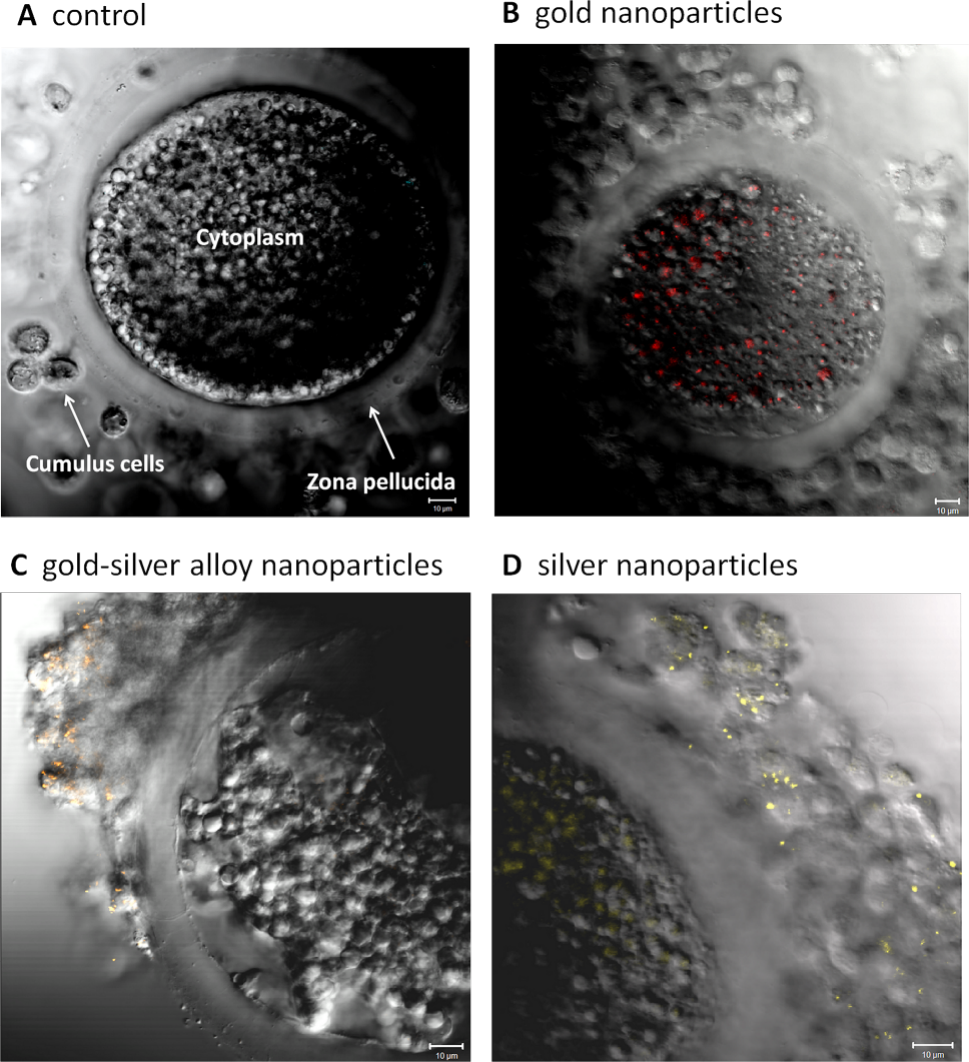
Representative laser scanning microscope images of porcine cumulus–oocyte complexes after 46 h co-incubation during in vitro maturation. (A) Negative control; (B) gold nanoparticles; (C) gold–silver alloy nanoparticles; (D) silver nanoparticles; bars = 10 micrometer. Reproduced with permission from [50]. Copyright 2014 Royal Society of Chemistry.

So far, only three studies have been published concerning the impact of nanoparticles on occytes. Hou and collegues examined the effect of titanium dioxide nanoparticles on isolated preantral rat follicles in vitro [75], while Hsieh et al. studied the cytotoxicity of CdSe quantum dots on the maturation of mouse oocytes, fertilization, and fetal development [76]. Both studies reported detrimental effects of the respective nanoparticles on oocyte maturation and further parameters, showing that female gametes are potentially at risk when exposed to nanoscale materials. Our own work investigated the response of porcine cumulus–oocyte complexes to BSA-coated gold, silver and gold–silver alloy nanoparticles in vitro [50]. Beyond the pure AuNP and AgNP the alloy particles were introduced in order to analyse in how far alloy formation alters the toxic potential of the pure metal nanoparticles. To estimate the effect of the tested nanoparticles on oocyte maturation the capability of the oocyte to reach the metaphase of the second meiosis and the expansion of the attached cumulus cells (Table 1) were examined. Gold nanoparticles of two significantly different sizes (6 nm versus 20 nm) and different surface modifications (BSA versus BSA+citrate) as well as different concentrations (10 µg/mL versus 30 µg/mL) showed no effect on the oocyte maturation (Figure 5). Despite the lack of toxicity, the particles were found to have been internalized in great numbers into the oocytes as observed by confocal microscopy (Figure 6). The directly adjacent cumulus cells incorporated gold nanoparticles to a substantially lesser degree. The gold–silver alloy particles displayed toxicity to oocytes depending on the silver molar fraction they contained. Up to 50% silver no significant adverse effects were found, while 80% silver lead to an almost complete arrest of oocyte maturation. The same applied to pure AgNP (Figure 5). Surprisingly, independent of their toxicity, silver containing particles preferentially associated to the cumulus cells rather than the oocyte as such (Figure 6).

**Figure 7:**
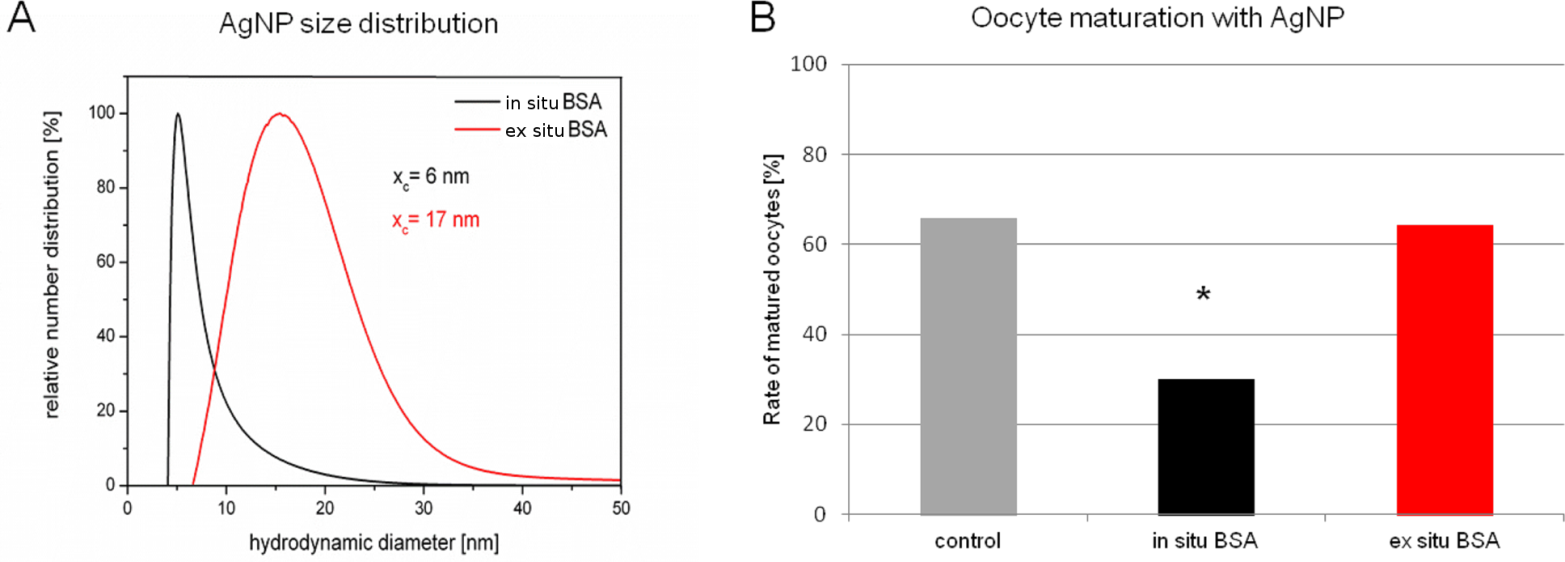
(A) Number weighted size distribution of AgNP in situ (red line) and ex situ (black line) conjugated to bovine serum albumin (BSA) as measured by disc centrifugation. x_c_ has been calculated by log-normal fitting. (B) Oocyte maturation rates after exposure to AgNP in situ or ex situ bioconjugated to BSA. *p < 0.05.

Even though there is no further literature which may be directly compared to our findings, with regard to toxicity our results agree with the general conception of cytotoxic effects excercised by gold and silver nanoparticles, which views silver as potentially more aggressive than gold [35]. The exact mechanisms by which silver nanoparticles inflict their damage is still a matter of debate. Although the toxicity seems to be driven by oxidation and inflammation [77], it is unclear whether silver in its nanoparticulate form is responsible for the toxic effects, as some studies claim [78], or whether they are solely caused by silver ions dissolving in the course of oxidation of the metal [20]. In our study silver ions proved to be equally toxic than alloy particles containing 80% of silver and pure AgNP pointing out that at least their toxic potential is similar. More recent and so far unpublished data seems to further confirm the hypothesis, that silver nanoparticle toxicity is mainly derived from the silver ions. In a small pilot study we compared the effects of silver nanoparticles, which are conjugated to BSA *“*in situ*”* and *“*ex situ*”* on oocyte maturation*.* In case of in situ bioconjugation silver nanoparticles are synthesized by laser ablation of a solid target in the presence of the biomolecule of choice [52,79]. The ex situ method is an alternative approach where the ablation site is physically separated from bioconjugation [80]. To this end laser ablation is carried out in a flow through reactor, while biomolecules are added at specified time delays. Innate to the in situ bioconjugation method is a distinct size quenching effect [81]. This leads to considerable smaller particles compared to the ex situ approach, as can be seen in Figure 7A. Interestingly, while the small in situ bioconjugated AgNP exibited a clear toxic effect on oocyte maturation as had been noted in earlier experiments [50], the larger ex situ produced BSA–AgNP displayed no toxicity at all (Figure 7B). As small nanoparticles possess a higher surface area, dissolution of Ag^+^ ions occurs to a greater extend than in case of larger particles. Therefore, if Ag^+^ ions are responsible for the observed effects, these should be more grave after exposure to small AgNP, as indeed they are. However, one has to be cautious to contribute the difference in toxic potential solely to the obvious difference in size. There might be other so far undected differences between the particles, which could have caused the described results, especially as silver nanoparticles keep evolving in biological media [82].

It seems noteworthy, that the oocytes showed no response after exposure to alloy particles containing 50% of silver, while the same silver concentration (3.3 mg/mL^−1^) and less has frequently been reported to have reprotoxic effects [78,83–85]. This suggests that incorporation of silver into an alloy structure can be used to control the toxicity of silver nanoparticles, which confirms findings observed after exposing bacterial cultures to gold–silver alloy nanoparticles [54].

Another interesting point in our studies was the above described difference in internalisation behavior between pure gold and silver containing nanoparticles (Figure 4). The reasons for that phenomenon can only be speculated about. Even though all particles were initially coated with BSA, they might have attracted a different set of “secondary” surface proteins, depending on the presence or absence of silver in the particles, once transferred into the biological medium. As these proteins define the biological identity of a particle [86], they might be responsible for the variations in particle uptake.

The described results confirm that in vitro maturation of oocytes represent a very sensitive system for the exploration of nanotoxicology in which even subtle effects can be visualized. The use of this test system should be increased in the future to gain a better understanding of possible influences of nanoparticle exposure on female reproduction.

### Effect of gold and silver nanoparticles on embryos

Within the field of nanoreprotoxicology embryo development is certainly the most intensively investigated area. Among those experiments many have been conducted by using gold and silver nanoparticles. The majority of this research focussed on piscine species, especially zebrafish, because such embryos can be obtained easily and in great numbers. A highly repeatable finding in these studies was the severe embryotoxicity of silver nanoparticles, while gold nanoparticles elicited hardly any adverse response [87–90] unless administered in very small sizes (<3 nm) [91–94]. In case of the latter it was shown that surface functionalisation had an additional influence on the severity and the characteristica of the observed toxicity [93–94]. Another interesting point raised was the impact of colloidal stability in the final exposure medium on the toxic potential of gold as well as silver nanoparticles showing a decrease in toxicity with increasing agglomeration, i.e., particle size [95–96]. Internalisation of nanoparticles into the embryos in a concentration dependent manner was also a consistent finding [83,90]. Oxidative stress was confirmed as the predominant mechanism responsible for toxic effects of AgNP on piscine embryos [97].

However, the results derived from marine species under conditions specific for external fertilisation and embryo development cannot simply be extrapolated to other species. This becomes apparent when reprotoxicological studies using chicken embryos, which were exposed in ovo to nanoparticles made from gold [98], silver [99–102], silver–palladium alloy [103] and silver–copper alloy [102] are considered. While gold nanoparticles remained consistently inert, even after application of silver containing nanoparticles, no abnormal development was observed, except a low-grade inflammation of the embryonic liver after exposure to AgCu alloy nanoparticles. Similar observations were made when administering gold and silver nanoparticles into pregnant mouse and rat dams respectively [44,104]. The reason for the apparent discrepancy between piscine and other species concerning the toxicity of silver-containing nanoparticles could be that the amount of nanoparticles which actually reached the embryo was a lot higher in the trials using piscine species. In these cases the particles were applied basically directly to the embryos in a environment of comparatively low complexity. Even though the initial doses in the chicken and rodent studies were comparatively high, the nanoparticles were first exposed to the contents of an egg or even to an entire different organism (the dam), which probably reduced the nanoparticle load of the embryo tremendously. In rats for instance it has been reported that of the AuNP dose applied to the mother by intravenous injection the foetus would take up approximately 0.0005–0.00006% of the particles [43]. This is supported by a study where murine preimplantation embryos were exposed in vitro, i.e., directly, to silver nanoparticles [105]. Similar to the trials using piscine embryos, the authors found abnormalities during preimplantation development like increased apoptosis at the blastocyst stage in conjuction with a reduced number of pups if nanoparticle exposed blastocysts where transferred to recipient dams. Therefore, when interpreting such data for its predictive value, the assessment of the exposure dose for its closeness to reality is a critical point. Interestingly, gold nanoparticles did not seem to trigger any toxicity in embryos regardless of dose [106].

**Figure 8:**
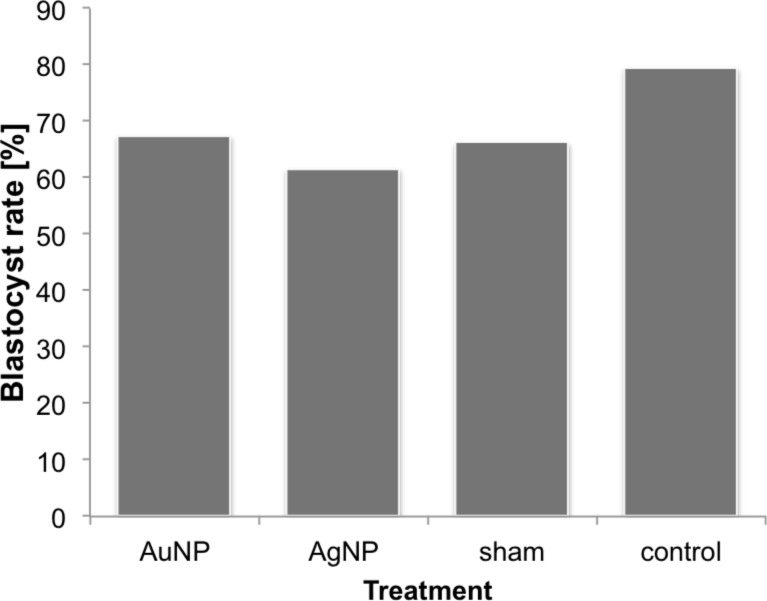
Blastocyst development rates after microinjection of nanoparticles into 2-cell-stage murine embryos (AuNP-injection, AgNP-injection, sham injection, handling control) (adapted from [51]).

In our own study, the effects of gold and silver nanoparticles on murine embryos was investigated [51]. The particles were microinjected into one blastomere of a 2-cell murine embryo, thus ensuring the delivery of small, i.e., realistic, but exact amounts of nanoparticles. The sister blastomere remained untreated allowing for an internal control. This increases the sensitivity of the test system by also pointing out sublethal effects, like the interference with cell division mechanisms, which has been described as a toxic effect of gold nanoparticles [107]. The administered dose of nanoparticles was calculated based on the assumption that an embryo would take up approximately 0.0004% of the particles [43] applied to the mother in a clinically relevant dosis [31,108]. The applied gold and silver nanoparticles did not elicit any effect (Figure 8). Neither the pre-implantation development of the embryos up to blastocyst stage was impaired, nor could a disregulation of various candidate genes for embryo development be found. Especially with regard to silver nanoparticles, this result might seem surprising at first. However, our results support the findings of the above mentioned in ovo and in vivo studies [44,98–99,104]. Our in vitro study used similar dosages as can be expected after in vivo exposure and we also obtained similar results. This further confounds the hypothesis that the effects seen in studies with direct embryonic exposure to silver nanoparticles derive from extremly high dosages of nanoparticles per embryo. Therefore, to improve the predictive value of future in vitro studies the experimental design should involve the testing of dosages realistic for in vivo exposure scenarios. However, to facilitate this, more biodistribution studies need to be performed, which firstly should also work with dosages and application routes appropriate for the kind of particle tested and secondly actually include reproductive organs as well as embryos into their examinations.

## Conclusion

The results described in this review with regard to the reprotoxicology of gold and silver nanoparticles permit the following conclusions: (i) Exposure of reproduction relevant cells to gold and silver nanoparticles after systemic administration has been proven. However, a considerable uncertainty exists concerning the particles properties once they arrived at their site of action. Especially the surface molecules, which likely define the bio-identity of the particles, will probably have changed after biodistribution compared to the pristine particles. Cellular internalisation depends on cell type as well as particle composition. Spermatozoa showed no evidence of particle uptake at all. Oocytes preferentially internalised pure gold nanoparticles, while gold–silver alloy particles as well as pure silver nanoparticles where mainly found in the cumulus cells surrounding the oocytes. (ii) Gold nanoparticles seem to be highly biocompatible, also in reproduction relevant settings. However, even gold nanoparticles were observed to be toxic to spermatozoa in a concentration dependent manner, in case the nanoparticles possess surface properties that allow direct contact with the sperm plasma membrane. Protein coronas seem to inhibit such contact. Gold nanoparticles also elicited a toxic response on embryos if applied in extremly small sizes in conjuction with high dosages (<2 nm, 10^14^ NP per embryo). (iii) Concerning oocytes and embryos silver nanoparticles are considerable more toxic than gold nanoparticles, with a clear dependency on the applied dosage. A clearly defined toxic threshold is difficult to determine though, as silver nanoparticle toxicity also depends on particle size as well as particle composition. The latter could distinctly been shown by employing gold–silver alloy colloids as model nanoparticles. The active components seem to be the Ag^+^ ions released from the particles, rather than the nanoparticles itself. Spermatozoa have been shown to be considerable more resistant towards silver nanoparticle derived toxicity, which might be explained by the unique metabolism spermatozoa feature compared to other cells.

Future research should aim to establish clear specifications which nanoparticle dose can be expected to be toxic under consideration of particle characteristics obtained under relevant biological conditions. Additionally, nanoparticle toxicity should not only be asssessed considering cell viability but also concerning functional aspects. To this purpose the investigation of nanotoxicology on reproductive cells provides an ideal tool.
